# Conformational Analysis of Uniformly ^13^C-Labeled Peptides by Rotationally Selected ^13^Cα-^13^CH_3_ Double-Quantum Solid-State NMR

**DOI:** 10.3390/molecules30030739

**Published:** 2025-02-06

**Authors:** David Middleton

**Affiliations:** Department of Chemistry, Lancaster University, Lancaster LA1 4YB, UK; d.middleton@lancaster.ac.uk; Tel.: +44-1524-594328

**Keywords:** rotational resonance, magic-angle spinning, biopharmaceuticals, amyloid

## Abstract

Peptides are an important class of biomolecules that perform many physiological functions and which occupy a significant and increasing share of the pharmaceutical market. Methods to determine the solid-state structures of peptides in different environments are important to help understand their biological functions and to aid the development of drug formulations. Here, a new magic-angle spinning (MAS) solid-state nuclear magnetic resonance (SSNMR) approach is described for the structural analysis of uniformly ^13^C-labeled solid peptides. Double-quantum (DQ) coherence between selective pairs of ^13^C nuclei in peptide backbone and side-chain CH_3_ groups is excited to provide restraints on (i) ^13^C–^13^C internuclear distances and (ii) the relative orientations of C–H bonds. DQ coherence is selected by adjusting the MAS frequency to the difference in the resonance frequencies of selected nuclear pairs (the rotational resonance condition), which reintroduces the dipolar coupling between the nuclei. Interatomic distances are then measured using a constant time SSNMR experiment to eliminate uncertainties arising from relaxation effects. Further, the relative orientations of C–H bond vectors are determined using a DQ heteronuclear local field SSNMR experiment, employing ^13^C–^1^H coupling amplification to increase sensitivity. These methods are applied to determine the molecular conformation of a uniformly ^13^C-labeled peptide, N-formyl-l-methionyl-l-leucyl-l-phenylalanine (fMLF). From just six distance and six angular restraints, two possible molecular conformations are determined, one of which is in excellent agreement with the crystal structure of a closely related peptide. The method is envisaged to a useful addition to the SSNMR repertoire for the solid-state structure determination of peptides in a variety of forms, including amyloid fibrils and pharmaceutical formulations.

## 1. Introduction

Peptides perform a wide range of different biological functions, including hormone signalling, pheromone communication and antimicrobial protection [1,2,3]. With current worldwide sales in excess of USD 70 billion, peptides are also an increasingly important class of biopharmaceuticals [1]. Knowledge of the three-dimensional molecular structure of a peptide is important if one is to understand its functional mechanism or fully characterize a therapeutic formulation. X-ray crystallography is usually the method of choice for structure determination, but there are instances in which peptide molecules do not produce diffracting crystals, such as when in amorphous formulations [2], biomembrane environments [3] or the binding sites of biomacromolecular targets [4].

Magic-angle spinning (MAS) solid-state NMR (SSNMR) spectroscopy is a powerful and flexible tool for the molecular structural analysis of solid or gel-like proteins and peptides, including those that lack long-range order and are inaccessible to diffraction methods. Peptides and proteins are readily amenable to isotopic labelling [5] (e.g., with ^13^C and ^15^N) to enhance NMR sensitivity, which, combined with the development of a repertoire of SSNMR methods [6,7], has supported the structural determinations of proteins comprising over 60 amino acid residues [8,9,10,11]. Further gains in sensitivity have been enabled by direct detection of ^1^H, with improved resolution provided by ultrafast probe technology achieving MAS frequencies of >110 kHz to reduce dipolar couplings and thereby extend the coherence lifetimes of protons in solids [12]. Proton resonances may also be observed by using sample perdeuteration followed by back-exchange of amide protons; the remaining weak ^1^H–^1^H dipolar couplings may be suppressed by sample spinning at relatively modest frequencies [13]. Restraints obtained by SSNMR measurements have underpinned the structural models of peptides in amyloid fibril assemblies, including the amyloid Aβ peptides associated with Alzheimer’s disease [14]. MAS SSNMR methods have also enabled the structure determination of membrane-embedded proteins and peptide channel assemblies [6,7].

SSNMR restraints on larger protein structures are usually extracted from chemical shift values [8], which are sensitive to backbone torsional angles defining local secondary structure [9], and from cross-peak intensities reporting long-range tertiary interactions between amino acids [10]. Additional restraints such as dipole tensor orientations can help to refine initial structural models [11]. Several hundred such restraints may be measured for a large protein, and a low precision of individual restraints may be tolerated because of the high combined power of all restraints. Shorter peptides yield far fewer restraints and so higher precision and accuracy are demanded of the limited data to determine the solid-state structure reliably. In these cases, restraints can take the form of internuclear distance ranges (±0.5 Å) and bond orientations. Complete structure determinations of short ^13^C,^15^N-labeled peptides have been enabled by measurements of distances and torsional angles defined by bonded H–C–C–H, H–C–N–H and N–C–C–N nuclei along the peptide backbone [15] (methodology reviewed in [16]).

This work introduces a new MAS SSNMR approach, which prepares selective double-quantum (DQ) coherences between non-bonded ^13^C nuclei in uniformly ^13^C-labeled solid peptides. The approach exploits the rotational resonance (RR) phenomenon [17,18,19] to generate DQ coherences between selected pairs of Cα and side-chain CH_3_ groups in peptides. The excited DQ coherences report ^13^C–^13^C distances and the relative orientations of C–H groups, which together serve as powerful constraints on peptide molecular conformation. RR occurs when the MAS frequency matches the difference in the resonance frequencies of pairs of nuclei (in this case, the Cα and CH_3_ resonance frequencies) or is a factor thereof [20]. For the selected nuclear pair, RR reintroduces the dipolar coupling between them that is usually averaged to zero by sample spinning at other frequencies [21]. This recoupling of a specific pair of nuclei can be exploited to generate selective DQ coherence, which is a doubly excited state that correlates the two nuclei. Practically, DQ coherence can be exploited by passing through a filter in the NMR pulse sequence to remove the signals from all other nuclei in a molecule, thereby enabling only the nuclear pair of interest to be observed. It will be shown that, by doing so, experiments can be designed that are sensitive to C–C distances and the relative orientations of C–H bonds for selected pairs of ^13^C nuclei. A two-dimensional (2D) experiment may be used to observe the resonance frequencies of the individual nuclei in the direct (horizontal) dimension (the single-quantum (SQ) dimension), and in the indirect (vertical) dimension, the DQ frequency is the sum of the individual resonance frequencies.

Cα and CH_3_ sites were chosen here because their resonances are often easy to assign and because relatively low MAS frequencies are required to match the separation of their resonance frequencies (around 6–7 kHz at the magnetic field of 16.4 T applied here). The method described is relevant to peptides containing the amino acids alanine, valine, leucine, isoleucine, methionine and threonine. It is stressed, however, that the method can in principle be applied to any pairs of ^13^C nuclei for which the RR condition can be met. To demonstrate the method, it is shown that just a few DQ coherences between methyl and Cα carbons in the uniformly labelled chemotactic tripeptide N-formyl-l-methionyl-l-leucyl-l-phenylalanine ([U-^13^C,^15^N]fMLF) are able to provide powerful restraints on the peptide conformation.

## 2. Results and Discussion

### 2.1. Selective Excitation of DQ Coherence for the ( [U-^13^C,^15^N]fMLF Peptide

**Figure 1:**
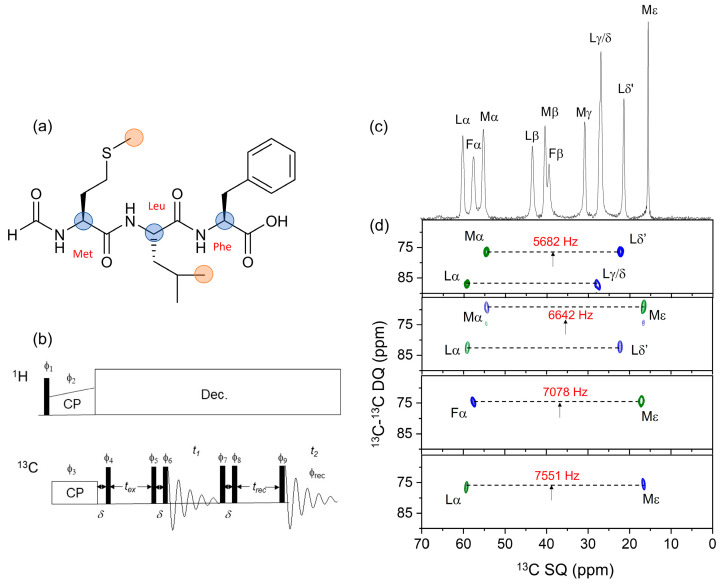
(**a**) Chemical structure of the fMLF peptide. Filled circles denote Cα (blue) and CH_3_ (orange) sites that were ^13^C–^13^C dipolar recoupled at rotational resonance. (**b**) Pulse sequence of the 2D ^13^C–^13^C DQSQRR experiment. All filled rectangles represent π/2 pulses. Signal is acquired over an 8-step phase cycle, with pulse phases ϕ_1_ = −y; ϕ_2_ = +x; ϕ_3_ = +x; ϕ_4_ = +x; ϕ_5_ = +x; ϕ_6_ = +y; ϕ_7_ = +x +y − x − y; ϕ_8_ = +y − x − y + x; ϕ_9_ = +x +x +x +x − x − x − x − x. The receiver phase ϕ_rec_ = +x +y − x − x − x − y +x + y. (**c**) One-dimensional ^13^C CP-MAS NMR spectrum of [U-^13^C,^15^N]fMLF showing Cα and aliphatic side-chain resonances only. (**d**) Two-dimensional ^13^C–^13^C DQSQRR spectra obtained using the pulse sequence in Figure 1b at MAS frequencies corresponding to the exact *n* = 1 rotational resonance condition with respect to Met Cα–Leu Cδ’ (5562 Hz), Met Cα–Met Cε (6642 Hz), Phe Cα–Met Cε (7078 Hz) and Leu Cα–Met Cα (7551 Hz). Note the shorthand: Lα, etc., refers to Leu Cα, etc. At ν_R_ = 6642 Hz, the chemical shift difference for Lα and Lδ’ is only 91 Hz away from *n* = 1 rotational resonance and therefore dipolar recoupling of these nuclear sites is observed. Arrows denote the carrier frequency. The ZQ excitation time, *t*_ex_, is 4 ms for each spectrum. Green and blue contours represent positive and negative intensities, respectively.

Figure 1a shows the chemical structure of fMLF, highlighting the positions of the atomic sites that will provide the structural restraints on the uniformly ^13^C-labeled peptide. Selective excitation of DQ coherence between methyl ^13^C and ^13^Cα nuclei is achieved at the *n* = 1 RR condition, whereby the rotor spinning rate, ν_R_, is set to the difference in resonance frequencies of the nuclear pair of interest, Δν_CC_, to reintroduce rotationally averaged ^13^C–^13^C dipolar couplings. With appropriate DQ filtration, the prepared DQ coherence can be observed selectively and all other signals eliminated from the SSNMR spectrum of the uniformly ^13^C-labeled peptide. To test the selectivity with which DQ coherence could be observed, we developed a two-dimensionally resolved adaptation of a pulse sequence described by Karlsson et al. [22,23], called 2D DQSQRR here (Figure 1b). In this experiment, after cross-polarization from ^1^H to ^13^C under the Hartmann–Hahn condition, the spins are allowed to evolve for a delay *δ* = 1/(2ν_R_) with proton decoupling and then a π/2 pulse is applied at a carrier frequency that is halfway between the frequencies of the ^13^C nuclei of interest. These steps store the Cα and CH_3_ magnetization as a difference polarization, I_z_–S_z_. A period, *t*_ex_, follows, during which the difference polarization is mechanically converted (i.e., by sample rotation) to zero quantum (ZQ) coherence at RR. A π/2–*δ*–π/2 pulse sequence is then applied to convert ZQ coherence to DQ coherence, which then evolves under a variable *t*_1_ delay for free evolution of the DQ coherence. The π/2–*t*_R_–π/2 sequence is repeated to convert DQ coherence back to ZQ coherence and is followed by delay *t*_rec_ = *t*_ex_ and a final π/2 pulse to produce observable magnetization. An eight-step phase cycle is applied, as described previously [22,23], to detect only the magnetization that has passed through the ZQ–DQ–ZQ filter. After 2D processing, the spectrum contains resonances from the Cα and CH_3_ spins at their chemical shifts (δ_Cα_ and δ_Me_) in the direct dimension and at the DQ frequency (δ_Cα_ + δ_Me_) in the indirect dimension. The final π/2 pulse of the sequence results in the observed CH_3_ resonances being inverted relative to the Cα resonances.

Figure 1c shows the 1D ^13^C cross-polarization (CP)-MAS NMR spectrum (aliphatic region) of a preparation of solid [U-^13^C,^15^N]fMLF diluted 1:4 with unlabelled peptide to reduce contributions from intermolecular couplings. Chemical shifts and peak widths, consistent with a microcrystalline sample, are given in Table S1 in the Supplementary Information. Figure 1d shows a series of 2D DQSQRR spectra obtained at different MAS frequencies and transmitter frequencies, selected to excite DQ coherence between different methyl and Cα sites in the peptide according to their proximity to the *n* = 1 RR condition. At MAS frequencies of 5682 Hz and 6642 Hz, DQ coherences are observed simultaneously for two pairs of Cα/CH_3_ sites (Figure 1d, top two panels). This is because (i) difference polarization may be generated for pairs of spins that resonate at different frequency offsets from the transmitter frequency (Supplementary Information, Figure S1) and (ii) DQ coherence may be excited even when the resonance frequencies do not match exactly the *n* = 1 rotational resonance condition (Figure S2).

### 2.2. Design of Experiments to Measure Internuclear Distances and C–H Bond Orientations

Experiments were designed to exploit the selective preparation of DQ coherence in order to measure distance and angle constraints on the peptide conformation. In principle, the 2D DQSQRR experiment can be adapted for this purpose, but the measurement times would be impracticably long. Importantly, the 2D spectra in Figure 1d confirmed that no additional DQ coherences involving the observed nuclei were excited. For example, the observed SQ signal for Mα (Figure 1d, top panel) is correlated only with Lδ’ and not with Lγ/δ. Likewise, in the same spectrum, the Lα SQ signal is correlated with Lγ/δ only, and not with Lδ’. The consequence of this observation is that measurements of interatomic distances and angles between specific nuclear pairs can be determined from the peak intensities in 1D spectra, requiring much shorter measurement times.

**Figure 2:**
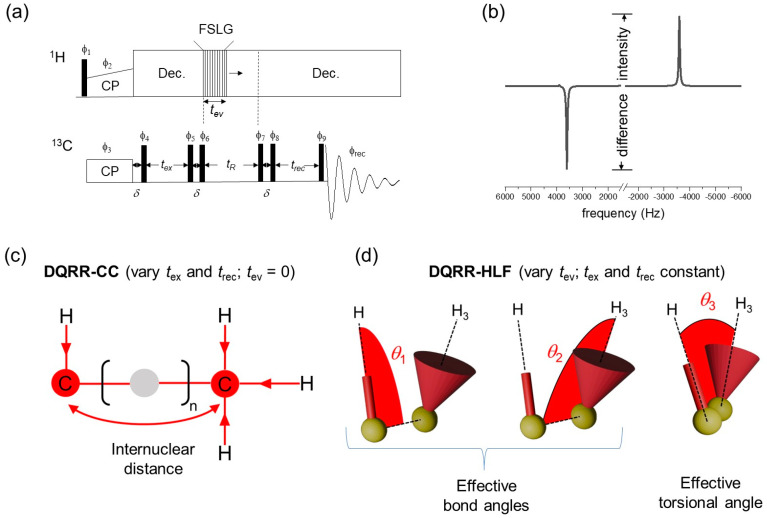
The basis of the 1D DQRR experiments for selective measurement of ^13^C–^13^C distances and ^13^C–^1^H bond orientations. (**a**) Basic pulse sequence. Phase cycling (ϕ_1_–ϕ_9_, ϕ_rec_) is as described in Figure 1b. FSLG = frequency-switched Lee–Goldburg sequence for ^1^H–^1^H decoupling. (**b**) How the difference intensity is measured from the observed NMR spectrum. (**c**) At *n* = 1 RR, dipolar interactions are recoupled between pairs of ^13^C nuclei if the separation is less than ~7 Å. In the DQRR-CC experiment, the difference intensity is modulated by varying *t*_ex_ and *t*_rec_. (**d**) In the DQRR-HLF experiment, t_ev_ is varied and the difference intensities are modulated according to the relative orientations of pairs of ^13^C–^1^H bonds (defined by angles θ_1_, θ_2_ and θ_3_).

Figure 2a presents the basic 1D pulse sequence used to generate DQ coherence at RR for measurements of distances and angles. Certain experimental parameters are controlled to obtain a series of spectra with resonance intensities that are sensitive to the distance-dependent dipolar coupling between the Cα and CH_3_ sites and to the relative orientations of the CαH and CH_3_ groups. In both cases, difference intensities measured from a series of spectra give the desired information (the definition of difference intensity is given in Figure 2b). The difference intensities are proportional to the difference polarization, I_z_–S_z_, at the end of the pulse sequence.

^13^Cα-^13^CH_3_ distance information is obtained by measuring difference intensities in a series of spectra recorded by varying the delays *t*_ex_ and *t*_rec_. This experiment is called DQRR-CC (Figure 2c). If *t*_ex_ = *t*_rec_ for all values of *t*_ex_, this is a variable time (VT) experiment (i.e., VT-DQRR-CC). The difference intensities follow the excitation of ZQ coherence and they increase and oscillate as a function of *t*_ex_ at a rate that depends on the dipolar coupling constant, *d*_CC_ (Figure S3a). The coupling constant is inversely proportional to the internuclear distance, *r*_CC_, according to the well-known relationship:

(1) $$d_{cc}=-\frac{\mu_{0}\gamma_{C}\gamma_{C}\hbar }{4\pi r_{CC}^{3}}$$

where $\gamma_{C}\;$ is the gyromagnetic ratio of ^13^C and all other terms have their usual meanings. In the VT-DQRR-CC experiment, the intensities are dampened by the influence of zero-quantum *T*_2_ relaxation, *T*_2_^ZQ^, which can be significant when distances are long and dipolar couplings are weak [24] (Figure S3a). *T*_2_^ZQ^ causes the NMR signal to decay exponentially, and its contribution to the observed response can be difficult to deconvolute from the dipolar-modulated oscillations that provide the distance information. The relaxation term is difficult to measure precisely, but a lower limit can be estimated from the sum of the peak widths at half-height [24]. To avoid uncertainty, a constant-time (CT) experiment (CT-DQRR-CC) was devised to eliminate the time-dependent effects of zero-quantum relaxation. In this version of the experiment, ZQ coherence is excited over a constant period, *t*_max_, whilst varying *t*_ex_ and *t*_rec_ such that *t*_ex_ + *t*_rec_ = *t*_max_. The difference intensities from *t*_ex_ = 0 to *t*_ex_ = *t*_max_ follows a sinusoidal profile that is a function only of *d*_CC_ and is symmetrical about the point when *t*_ex_ = *t*_rec_.

A second variation of the experiment in Figure 2a was developed to provide C–H bond orientational restraints. In this experiment, the selectively prepared ^13^C–^13^C DQ coherence is allowed to evolve for one rotor period under the influence of the heteronuclear local fields generated by the bonded protons. This experiment is called DQRR-HLF and the measured difference intensities report on the evolution of DQ coherence (Figure 2d). Non-selective MAS SSNMR experiments of this type have been used to measure H–C–C–H bond orientations in directly bonded four-spin systems [25]. An advantage of the experiment here is that the carbon sites do not need to be directly bonded and can be selected simply by adjusting the MAS frequency to the appropriate rotational resonance condition. The principle is to allow ^13^C–^13^C DQ coherence to evolve over one cycle of sample rotation, *t*_R_, whilst simultaneously applying proton homonuclear decoupling for time *t*_ev_. The DQ coherence is dephased by the local proton field over the first half of the sample rotation cycle and rephased over the second half of the rotation cycle and then converted to observable magnetization. A series of spectra are recorded by varying *t*_ev_ from zero to a maximum of *t*_R_, and a plot of ^13^C difference intensities against *t*_ev_/*t*_R_ is sensitive to the relative orientations of the C–H bonds (Figure S3b). CH_3_ groups typically undergo rapid rotation and so the spin system is approximated as a C–H bonded pair (i.e., C–H_3_) in which the position of the single proton is the average of the three methyl proton coordinates. Three angles, *θ*_1_, *θ*_2_ and *θ*_3_, define the relative orientations of the C–H and C–H_3_ groups (Figure 2d). The effective ^13^C–^1^H dipolar coupling constant (3800 Hz) is approximately 1/3 that of an actual C–H bonded pair (11,500 Hz) because of dynamic averaging. Restraints on the relative orientations are obtained by comparing the experimentally measured intensities with numerically simulated curves for different combinations of *θ*_1_, *θ*_2_ and *θ*_3_.

A limitation of the DQRR-HLF experiment is the potentially poor sensitivity of the measured intensities to different bond orientations. This is a consequence of the scaling of the C–H_3_ dipolar coupling by rotation of the CH_3_ group. To illustrate this point, Figure S4 (left) shows plots of simulated difference intensities for the DQRR-HLF experiment as a function of *t*_ev_ for all possible angle combinations (0° ≤ *θ*_1_ ≤ 180°; 0° ≤ *θ*_2_ ≤ 180°; 0° ≤ *θ*_3_ < 360°) at three MAS rates. The simulated curves span a rather narrow range, which confirms that the sensitivity of the experimental measurements to different angle combinations is limited when using this experiment. An adaptation to the pulse sequence was therefore made (Figure S5) to effectively amplify the dipolar couplings by a factor of 2, according to the principle of Hong et al. [26]. Simulated curves for the amplified experiment indicate that the angular sensitivity is improved, particularly at higher MAS frequencies (Figure S4, right).

### 2.3. Measurement of Internuclear Distances and C–H Bond Orientations in the ([U-^13^C,^15^N]fMLF Peptide

**Figure 3:**
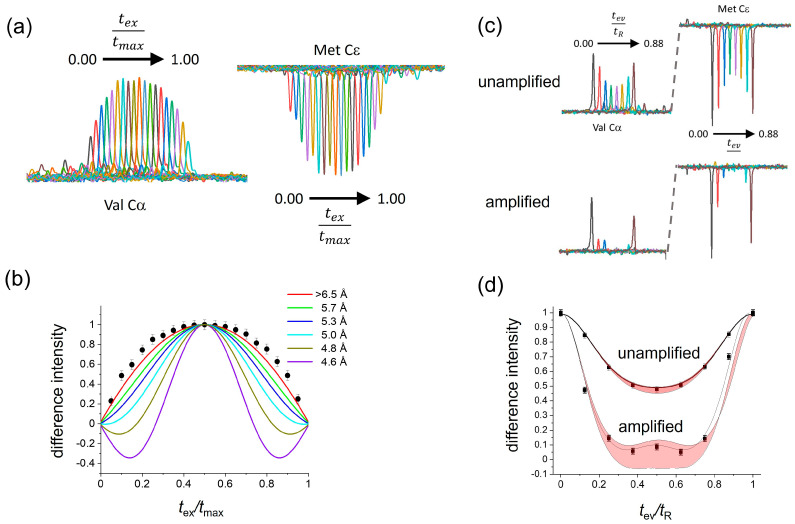
Determination of the Leu Cα–Met CεH_3_ relative orientations and ^13^Cα–^13^Cε distance in solid [U-^13^C,^15^N]fMLF. The MAS frequency was set to the *n* = 1 RR condition for Leu Cα and Met Cε (ν_R_ = 7551 Hz). (**a**) Series of spectra obtained in the CT-DQRR-CC experiment by varying *t*_ex_ and *t*_rec_. (**b**) Difference intensities (filled circles) measured from the spectra in (**a**) and simulated curves for different internuclear distances, *r*_CC_. Error bars represent the level of the noise. (**c**) Series of spectra obtained using the DQRR-HLF experiment, by varying *t*_ev_ up to the duration of one rotor cycle, *t*_R_, and maintaining *t*_ex_ and *t*_rec_ at 4 ms. The top spectra were obtained without dipolar amplification using the pulse sequence in Figure 2a, and the bottom spectra were obtained with dipolar amplification using the pulse sequence in Figure S5. (**d**) DQ evolution over one rotor cycle. Filled circles denote the DQ-filtered difference intensities measured from the amplified and unamplified experiments. The line of best fit (dotted line) is shown on the amplified data set. The red shaded areas bounded by the solid lines represent the range of variability of the difference intensities to all possible C–H and CH_3_ orientations.

Figure 3 shows the results of applying the CT-DQRR-CC and DQRR-HLF experiments to Met CεH_3_ and Leu CαH groups of fMLF. DQ coherence between the ^13^C sites of these groups was selected at *n* = 1 RR by setting the MAS frequency to 7551 Hz. All experimental measurements of difference intensities had associated errors determined by the level of the noise (shown as error bars in Figure 3 and in the Supplementary Figures). Hence, each set of data was considered to have upper and lower limits.

The CT-DQRR-CC experiment was used to determine the distance between Met Cε and Leu Cα. The measured difference intensities as a function of *t*_ex_/*t*_max_ were compared with numerically simulated curves for different values of the ^13^C–^13^C dipolar coupling constant (see Methods for details). All simulated curves falling within the upper and lower limits of the data, defined by the error bars, were considered to be consistent with the data. Accordingly, the measured difference intensities correspond to a distance of >6.5 Å between Met Cε and Leu Cα (Figure 3a,b). By contrast, the VT-DQRR-CC experiment cannot distinguish between C–C distances of between 4.5 Å and 6.5 Å because of uncertainties in the value of *T*_2_^ZQ^ (Figure S6) and hence was not used further.

The orientations of the Leu CαH and Met CεH_3_ groups were investigated using the unamplified and amplified DQRR-HLF experiments. The unamplified experiment is virtually insensitive to the different orientations (Figure 3c(top),d). However, by comparing the amplified DQRR-HLF data (difference intensities vs. *t*_ev_/*t*_R_) with simulated curves for different values of *θ*_1_, *θ*_2_ and *θ*_3_, approximately 50% of all the possible Cα–H and Cε–H_3_ bond orientations can be eliminated for being inconsistent with the data (Figure 3c, bottom, Figure 3d and Figure S7). The remaining combinations of *θ*_1_, *θ*_2_ and *θ*_3_ that provide consistency between the simulated curves and the DQRR-HLF data serve together with the C–C distance as restraints on the side-chain conformation of Met in the peptide.

Further restraints on the entire peptide conformation were obtained using the CT-DQRR-HLF and DQRR-CC experiments to report on interatomic distances and C–H orientations for Met CαH–Leu Cδ_1_H_3_, Leu CαH–Leu Cδ_1_H_3_, Met CαH–Met CεH_3_, Phe CαH–Leu Cδ_1_H_3_ and Phe CαH–Met CεH_3_ (Figure S8 and Table 1). The RR conditions for each spin pair are summarized in Table S2. A total of 12 sets of measurements (6 distances and 6 angles) was obtained.

### 2.4. Conformational Restraints on the ( [U-^13^C,^15^N]fMLF Peptide

**Figure 4:**
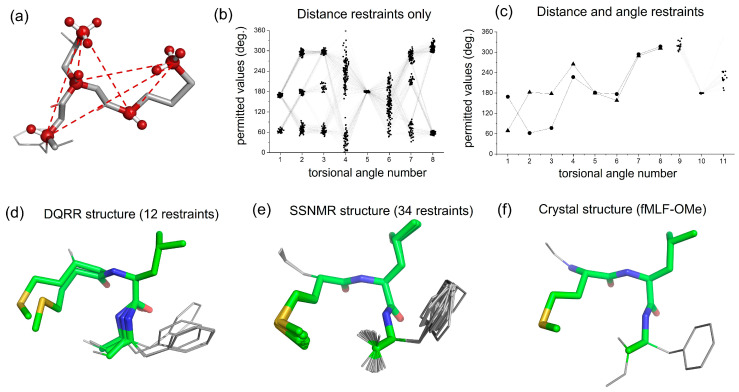
Conformational analysis of fMLF from the DQRR distance and angle restraints. (**a**) An unrestrained random structure of fMLF, highlighting the 6 selective ^13^C–^13^C DQ coherences forming the basis for the structural restraints. (**b**) Combinations of peptide side-chain and main-chain torsional angles 1–8 that are consistent with the 6 distance restraints only. Connecting lines represent individual peptide conformations. The torsional angle numbers are defined in Figure S9 and explained in Table S3. Angles 9 and 10 are omitted for clarity. (**c**) Combinations of peptide torsional angles that are consistent with all restraints (see also Table 2). Two conformations are represented by triangles (conformer 1) and circles (conformer 2). (**d**) Peptide molecular conformations (conformer 1 and conformer 2) corresponding to the restrained torsional angles in (**c**) and Table 2. (**e**) Molecular conformations of fMLF determined previously from SSNMR restraints (PDB 1Q7O). (**f**) Molecular conformation of C-terminal methoxy fMLF [28].

The distance and angle measurements were assessed for their ability to restrain the fMLF peptide in a single unambiguous conformation or ensemble of closely related conformations (Figure 4a). This was achieved by performing a conformational grid search of the fMLF molecule, starting from an initial unrestrained model constructed from standard bond lengths and angles and then randomly varying torsional angles across the main chain and side chains. The grid search included a probability density function derived from libraries of statistical data on preferred torsional angles of amino acids in peptides [27]. Thus, the backbone angles ϕ and ψ were confined to values predicted by Ramachandran analysis, and angles χ_1_, χ_2_, etc., of amino acid side chain rotamers typically have gauche(+) (χ_n_ ~ +60°), gauche(−) (χ_n_ ~ −60°) and trans (χ_n_ ~ 180°) conformations. Molecular coordinates of 5 × 10^6^ peptide conformations were generated (Figure S9), of which only 166 were consistent with the distance ranges determined by DQRR-CC (Figure 4b). There was nevertheless considerable variation across these remaining conformations and so the angular constraints were necessary to reduce the uncertainties. For each conformation, DQ evolution curves were simulated for the relative orientations of Met CαH–Leu Cδ_1_H_3_, Leu CαH–Leu Cδ_1_H_3_, Met CαH–Met CεH_3_, Phe CαH–Leu Cδ_1_H_3_ and Phe CαH–Met CεH_3_ and compared with the experimental DQRR-HLF data. After applying all 16 restraints, just two closely related conformers (1 and 2) remained (Figure 4c,d). It should be noted that no energy calculations were performed to restrain the peptide conformation.

### 2.5. Comparison with X-Ray Crystallography

To assess the reliability of the SSNMR restraints, the peptide conformers determined here (Figure 4c,d) were compared with previously determined conformations of fMLF. There is good agreement between one of the conformations reported here (conformer 1) and a model obtained earlier from almost three times as many SSNMR restraints (Figure 4e and Table 2) [26]. Conformer 1 also agreed closely with the crystal structure of the related peptide, fMLF-OMe (Figure 4f and Table 2) [29]. Hence, for this peptide, the new SSNMR approach described here provides reliable structural restraints that compare favourably with other standard SSNMR methods and with X-ray crystallography. Conformer 2 deviates more from the crystal structure at the N-terminus but is in closer agreement toward the C-terminus (Table 2). It is probable that this conformer could be eliminated by measuring a further restraint, such as the distance between Phe Cα and Met Cγ, but this was not performed here.

### 2.6. Limitations of the Approach

Despite the good agreement with previous results, there are experimental variables that limit the precision and accuracy of the data obtained here, and for which there is scope for further improvement. First, typical data sets, such as those shown in Figure 3, require 24–48 h measurement times to achieve the signal-to-noise reported. The level of the noise introduces uncertainty in the measured peak intensities as reflected in the error bars. A trade-off between the acceptable precision, practicable data collection time and the amount of sample available must therefore be considered. Second, the measurements were performed at a magnetic field strength of 16.4 MHz, at which the frequency difference between the Cα and CH_3_ resonances (and hence MAS frequencies required) are on the order of 5–8 kHz. Lower magnetic field strengths may reduce signal to noise and also compromise the selectivity of DQ excitation. In this regard, we have found that when lower MAS frequencies are required to achieve RR (as would be needed at lower magnetic fields), unwanted coherences can be observed in the DQ filtered spectra. We suggest that these experiments are best performed at magnetic fields of 14.1 T or higher. Finally, the widths of the resonance lines can also introduce uncertainties, particularly when peaks are broadened due to structural inhomogeneity. One consequence of this is that not all crystallites in a sample may experience exact RR at one MAS frequency, and so data such as that reported in Figure 3 may reflect both on-resonance and off-resonance components. Greater accuracy may be achieved for highly ordered crystalline materials or else off-resonance contributions may be considered the numerical simulations.

## 3. Methods and Materials

### 3.1. Preparation of fMLF for SSNMR

[U-^13^C,^15^N]fMLF was obtained from CortecNet (Les Ulis, France), and unlabelled peptides were prepared by solid-phase synthesis. A 4:1 mixture of unlabelled:labelled peptides (30 mg total) was added to the minimum volume of 2-propanol required for dissolution, and the solvent was allowed to slowly evaporate overnight in a drying oven at 35 °C. The remaining solvent was removed from the resultant precipitate by a syringe needle and blotting with tissue paper, and the solid was dried under vacuum. The solid (approximately 20 mg dry weight) was packed by centrifugation into a 3.2 mm zirconia rotor containing a space-filling plug at the bottom, to ensure that all material was contained in the centre of the rotor. The NMR peaks were quite narrow (Table S1), suggesting that the solid peptide was microcrystalline, but no further analysis of the crystal morphology was performed.

### 3.2. Preparation and Characterization of Med43-50 Fibrils

The Med43-50 peptide was prepared by solid-phase synthesis. Fibrils were prepared as follows. The peptide was subjected to three dissolution-evaporation cycles with hexafluoroisopropanol (HFIP) to break up any initial aggregates. The peptide was then dissolved in dimethylsulfoxide (DMSO) and added to double-distilled H_2_O to a final DMSO concentration of 10% (*v*/*v*) and a peptide concentration of 1 mg/mL. The solution was incubated with agitation at room temperature for 5 d, and insoluble fibrils were harvested by spinning down in a benchtop centrifuge for 30 min. A fibrillar morphology was confirmed by transmission electron microscopy using negative staining (4% uranyl acetate). Suspensions of the peptide aggregates (10 μL) were loaded onto carbon-coated copper grids and visualized on a Tecnai 10 electron microscope (Thermo Fisher Scientific, Waltham, MA, USA) at 100 kV.

### 3.3. NMR Analysis

Spectra were obtained on a Bruker Avance 700 instrument (Billerica, MA, USA) operating at a frequency of 176.03 MHz for ^13^C. Samples were spun at MAS rates of 5–8 kHz, and CP-MAS NMR spectra were obtained by averaging between 256 and 4096 transients, depending on the experiment. The global experimental parameters were a 3.0 μs ^1^H excitation pulse, 2 ms Hartmann–Hahn cross polarization at a proton radiofrequency field of 65 kHz, SPINAL-64 proton decoupling [17] at a field of 85 kHz during signal acquisition, and a 2 s recycle delay. All measurements were performed at ambient temperature.

The 2D DQSQRR spectra were obtained with 128 t_1_ increments and phase sensitivity in the indirect dimension achieved using the States-TPPI method. The 1D DQRR-CC experiment employed the pulse sequence given in Figure 1b. The ZQ excitation time, *t*_ex_, was varied from 1 ms to a maximum of 30 ms, during which heteronuclear decoupling was applied at a proton field of 85 kHz. The 1D DQRR-HLF experiments (Figure 1b and Figure S4) employed homonuclear decoupling of protons using the frequency-switched Lee–Goldburg sequence at a proton field of 100 kHz [18].

### 3.4. Computational Methods

Simulated curves were generated for molecular geometries of fMLF covering the entire conformational space of the peptide. The curves were compared with the NMR measurements, and a particular geometry was accepted if the corresponding simulated curve fell within the limits of the data. The general process for obtaining the restraints is described in the following.

An initial structural model of the peptide (including protons) was built in the PyMOL Molecular Graphics System (academic license; http://pymol.org/academic, accessed on 23 January 2025; Schrodinger, LLC. 2010, New York, NY, USA), utilizing standard bond lengths and angles to calculate the atomic coordinates. The main chain torsional angles, ϕ and ψ, for each amino acid residue, conformed to a β-sheet geometry in the initial model. The side-chain torsional angles (χ_1_, χ_2_, etc.) in the initial model took the default values defined by PyMOl. The initial atomic coordinates were input into a custom-written C program for the conformational grid search. In the grid search, each of the side-chain and main-chain torsional angles (Met [χ_1_,χ_2_,χ_3_,ϕ,ψ], Leu [χ_1_,χ_2_,ϕ,ψ] and Phe [χ_1_,ϕ,ψ]) was assigned a random value between 0° and 360° and a series of matrix rotations was performed on the initial coordinates, using the torsional angles as the rotational matrix elements. The values of the randomly generated torsional angles were assigned according to the probabilities of their occurrence in protein structures, with probabilities weighted according to a normal (Gaussian) distribution about the means (Table S3). For example, values of angle $\chi_{3}$ of Met were distributed about means of 311° (3°), 60° (5°) or 169° (3°), where the values in brackets are the standard deviations. A total of 5 × 10^6^ sets of angles were generated, and the distributions of values for each angle are shown in Figure S9. From these angles, 5 × 10^6^ different sets of peptide coordinates were generated, each representing a different molecular conformation. For each conformation, the coordinates of the appropriate atoms were taken to calculate the C–C distance (*r*_CC_), corresponding dipolar coupling (*d*_cc_) and C–H/C–H_3_ bond orientations (*θ*_1_, *θ*_2_ and *θ*_3_, defined in Figure 2d). These variables were the input parameters for the simulations of the NMR response in the DQRR-HLF and DQRR-CC experiments.

Simulations of the difference magnetization, <Iz–Sz>, observed in the DQRR-HLF and DQRR-CC experiments were carried out in the SIMPSON software environment (SIMPSON 4.1.1; https://inano.au.dk/about/research-centers-and-projects/nmr/software/simpson, accessed on 23 January 2025). The initial density matrix, ρ(0), denoted as Inz in the SIMPSON input file, represented both spins at equilibrium in order for the effects of pulse frequency offsets to be simulated from the beginning of the pulse sequences. The input parameters included the MAS frequency, the Euler angles describing the orientation of C–H and C–H_3_ bond vectors (angles θ_1_, θ_2_ and θ_3_ in the main text), and the ^13^C–^13^C and ^13^C–^1^H dipolar coupling constants. The SIMPSON code (see Figures S10–S12 for examples) was generated and invoked by a bespoke C program which generated the atomic coordinates for 5 × 10^6^ peptide conformations and calculated the C–H geometric parameters that were needed for input into SIMPSON.

## 4. Conclusions and Outlook

SSNMR is a useful method for the structural analysis of peptides and proteins in the solid state. Secondary structure information can be provided by chemical shift analysis using the program TALOS to establish an empirical relationship between ^13^C, ^15^N and ^1^H chemical shifts and backbone torsion angles [9]. Chemical shift computations using DFT approaches are also valuable, but the calculations may require knowledge of intermolecular contributions such as crystal packing effects [19,30]. Precise restraints on interatomic distances and on bond orientations are therefore important for defining the three-dimensional structures of short peptides. The new approach reported here can access both distance and angular information from the same basic NMR pulse sequence and can select coherences between non-bonded ^13^C pairs by simply adjusting the MAS frequency to the appropriate RR condition.

**Figure 5:**
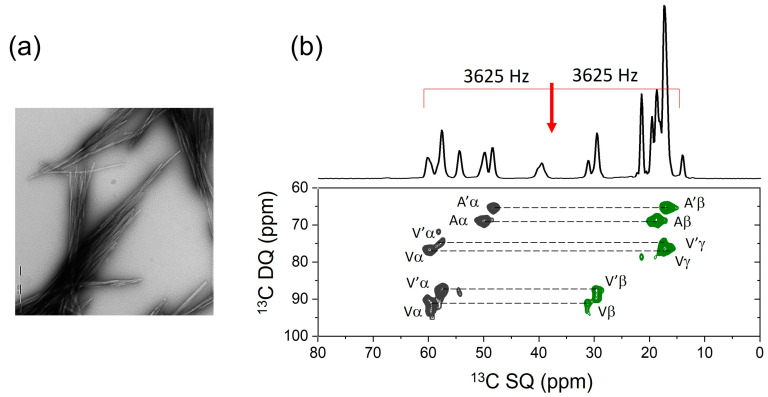
Preliminary SSNMR analysis of the peptide [U-^13^C-FVA]Med43-50. (**a**) Negative-stain TEM image of the peptide fibrils. (**b**) Two-dimensional DQSQRR SSNMR spectrum of the peptide fibrils at a MAS frequency of 7250 Hz, which is close to, but does not correspond exactly to, the *n* = 1 RR condition for any pair of observed nuclei. The red arrow signifies the spectrometer carrier frequency. The doubling of resonances (Aα, A’α, Aβ, A’β, etc.) is attributed to the polymorphism of the fibrils.

This approach is envisaged to be a valuable addition to the NMR toolkit for the structural analysis of peptides and small proteins in various environments and preparations. SSNMR has proved to be particularly useful for supplying restraints on the molecular architectures and interactions of amyloid fibrils, which are the fibrous nanoparticles of hydrogen-bonded peptide β-strands that are associated with human disease and also perform functional roles [29,31]. SSNMR measurements of chemical shifts and dipolar couplings have resolved the secondary, tertiary and quaternary structural details for amyloid fibrils formed by several proteins and peptides, including the Alzheimer’s amyloid-β peptides [32]. At present, 47 structural models of amyloid, derived from SSNMR, are deposited in the Protein Data Bank. SSNMR restraints generally do not give details of the amino acid side-chain conformations in amyloid fibrils, yet this information is relevant to the interactions of fibrils with drugs and accessory molecules [31,33]. The SSNMR approach here can offer fine details on side-chain conformations that would complement the broader structural information provided by other SSNMR methods and, increasingly, by cryo-electron microscopy [34]. To illustrate this point, Figure 5 shows the 2D DQSQRR SSNMR spectrum of fibrils formed by a peptide of sequence NH_2_-FGSVQFVA-COOH, representing the amyloidogenic C-terminal region of the 50-amino acid aortic amyloid polypeptide, medin (Med43-50). The peptide is uniformly ^13^C-labeled at the C-terminal residues Phe, Val and Ala. Although no structural measurements are reported here, the spectrum demonstrates that with judicious selection of the RR condition and using the CT-DQRR-CC and DQRR-HLF experiments, DQ coherences are excited, from which it will be possible to gain information about the Phe and Val side-chain conformations in addition to the backbone torsional angles. As a further example, our new SSNMR approach may provide structural details on hydrophobic peptides, which are difficult to crystallize for X-ray analysis and which may have a tendency to self-associate [35]. In this regard, the methods may be useful for characterizing formulations of hydrophobic peptide drugs [36].

## Figures and Tables

**Table 1:** Summary of SSNMR conditions and measurements using the CT-DQRR-CC experiments. Errors are given in parentheses.

CαH	CH_3_	ν_R_ (Hz)	*t*_max_ (ms)	*r*_CC_ (Å)
Met	Met	6642	20	4.8 (0.2)
Met	Leu	5682	16	>5.0
Leu	Met	7551	30	>6.5
Leu	Leu	6533	10	3.0 (0.2)
Phe	Met	7078	20	5.6 (0.2)
Phe	Leu	6107	25	>6.5

**Table 2:** Summary of torsional angles for solid fMLF determined using the SSNMR restraints here, from previous SSNMR restraints and by X-ray crystallography.

Torsional Angle ^a^	Value (Degrees)			
	Conformer 1 ^b^	Conformer 2 ^b^	Previous SSNMR ^c^	X-Ray ^d^
1	69	169	87	77
2	182	62	157	173
3	178	77	153	180
4	265	227	282	243
5	180	181	177	175
6	158	177	145	167
7	289	294	302	300
8	312	317	305	302
9	301	311	316	310
10	179	179	180	176
11	211	172	195	204

^a^ The numbering system for the torsional angles is given in Figure S9. ^b^ From the 16 restraints obtained here. ^c^ From 34 SSNMR restraints [26]. ^d^ From the X-ray crystal structure of fMLF-OMe.

## Data Availability

Available from the author on request.

## Supplementary Materials

The following supporting information can be downloaded at https://www.mdpi.com/article/10.3390/molecules30030739/s1. Table S1: Summary of ^13^C chemical shifts and full widths at half height for solid [U-^13^C,^15^N]fMLF; Table S2: Summary of the ^13^C–^13^C DQ coherences selected for solid [U-^13^C,^15^N]fMLF and corresponding MAS frequencies for the distance and angle restraints; Table S3: Summary of statistically restricted torsional angles used in the conformational grid search; Figure S1: Simulated peak intensity profiles of a dipolar-coupled I–S spin pair showing the effect of varying the resonance frequencies relative to the transmitter/carrier frequency at 0 Hz; Figure S2: Simulated peak intensity profiles of a dipolar-coupled I–S spin pair, showing the effect of frequency offsets from the *n* = 1 rotational resonance condition; Figure S3: Simulated difference intensity profiles for the DQRR-CC and DQR-HLF experiments; Figure S4: Sensitivity of the DQRR-HLF experiment to the relative orientations of C–H and –CH_3_ groups at three difference MAS frequencies; Figure S5: Pulse sequence for the amplified DQRR-HLF experiment; Figure S6: Measurement of the distance-dependent ^13^Cα–^13^Cε dipolar coupling between Leu and Met of solid [U-^13^C,^15^N]fMLF, using the VT-RRDQ-CC experiment; Figure S7: Allowed Cα–H and CεH_3_ orientations of Leu and Met in solid [U-^13^C,^15^N]fMLF, consistent with the DQRR-HLF measurements; Figure S8: Analysis of the Met side chain conformation in solid [U-^13^C,^15^N]fMLF peptide; Figure S9: Summary of statistically weighted probabilities of torsional angles that were varied randomly in the conformational grid search. Figures S10–S12: Example SIMPSON input files used for numerical simulations of the NMR data. Data sets and NMR pulse sequences are available from the author on request.

## References

[1] Wang L., Wang N.X., Zhang W.P., Cheng X.R., Yan Z.B., Shao G., Wang X., Wang R., Fu C.Y. (2022). Therapeutic peptides: Current applications and future directions. Signal Transduct. Target. Ther..

[2] Weers J.G., Miller D.P. (2015). Formulation Design of Dry Powders for Inhalation. J. Pharm. Sci..

[3] Malmsten M. (2016). Interactions of Antimicrobial Peptides with Bacterial Membranes and Membrane Components. Curr. Top. Med. Chem..

[4] Worm D.J., Els-Heindl S., Beck-Sickinger A.G. (2020). Targeting of peptide-binding receptors on cancer cells with peptide-drug conjugates. Pept. Sci..

[5] Lian L.Y., Middleton D.A. (2001). Labelling approaches for protein structural studies by solution-state and solid-state NMR. Prog. Nucl. Magn. Reson. Spectrosc..

[6] Cady S.D., Schmidt-Rohr K., Wang J., Soto C.S., DeGrado W.F., Hong M. (2010). Structure of the amantadine binding site of influenza M2 proton channels in lipid bilayers. Nature.

[7] Lange A., Giller K., Hornig S., Martin-Eauclaire M.F., Pongs O., Becker S., Baldus M. (2006). Toxin-induced conformational changes in a potassium channel revealed by solid-state NMR. Nature.

[8] Wishart D.S., Case D.A. (2001). Use of chemical shifts in macromolecular structure determination. Nucl. Magn. Reson. Biol. Macromol..

[9] Shen Y., Delaglio F., Cornilescu G., Bax A. (2009). TALOS plus: A hybrid method for predicting protein backbone torsion angles from NMR chemical shifts. J. Biomol. NMR.

[10] Kraus J., Sarkar S., Quinn C.M., Polenova T., Webb G.A. (2021). Solid-state NMR spectroscopy of microcrystalline proteins. Annual Reports on NMR Spectroscopy.

[11] Franks W.T., Wylie B.J., Schmidt H.L.F., Nieuwkoop A.J., Mayrhofer R.M., Shah G.J., Graesser D.T., Rienstra C.M. (2008). Dipole tensor-based atomic-resolution structure determination of a nanocrystalline protein by solid-state NMR. Proc. Natl. Acad. Sci. USA.

[12] Nishiyama Y., Hou G.J., Agarwal V., Su Y.C., Ramamoorthy A. (2022). Ultrafast Magic Angle Spinning Solid-State NMR Spectroscopy: Advances in Methodology and Applications. Chem. Rev..

[13] Barbet-Massin E., Pell A.J., Retel J.S., Andreas L.B., Jaudzems K., Franks W.T., Nieuwkoop A.J., Hiller M., Higman V., Guerry P. (2014). Rapid Proton-Detected NMR Assignment for Proteins with Fast Magic Angle Spinning. J. Am. Chem. Soc..

[14] Lührs T., Ritter C., Adrian M., Riek-Loher D., Bohrmann B., Döeli H., Schubert D., Riek R. (2005). 3D structure of Alzheimer’s amyloid-β(1-42) fibrils. Proc. Natl. Acad. Sci. USA.

[15] Rienstra C.M., Tucker-Kellogg L., Jaroniec C.P., Hohwy M., Reif B., McMahon M.T., Tidor B., Lozano-Pérez T., Griffin R.G. (2002). *De novo* determination of peptide structure with solid-state magic-angle spinning NMR spectroscopy. Proc. Natl. Acad. Sci. USA.

[16] van der Wel P.C.A. (2021). Dihedral Angle Measurements for Structure Determination by Biomolecular Solid-State NMR Spectroscopy. Front. Mol. Biosci..

[17] Fung B.M., Khitrin A.K., Ermolaev K. (2000). An improved broadband decoupling sequence for liquid crystals and solids. J. Magn. Reson..

[18] van Rossum B.J., Forster H., deGroot H.J.M. (1997). High-field and high-speed CP-MAS ^13^C NMR heteronuclear dipolar-correlation spectroscopy of solids with frequency-switched Lee-Goldburg homonuclear decoupling. J. Magn. Reson..

[19] Middleton D.A., Griffin J., Esmann M., Fedosova N.U. (2023). Solid-state NMR chemical shift analysis for determining the conformation of ATP bound to Na,K-ATPase in its native membrane. RSC Adv..

[20] Raleigh D.P., Levitt M.H., Griffin R.G. (1988). Rotational resonance in solid-state NMR. Chem. Phys. Lett..

[21] Dusold S., Sebald A., Webb G.A. (2000). Dipolar recoupling under magic-angle spinning conditions. Annual Reports on NMR Spectroscopy.

[22] Karlsson T., Edén M., Luthman H., Levitt M.H. (2000). Efficient double-quantum excitation in rotational resonance NMR. J. Magn. Reson..

[23] Karlsson T., Hughes C.E., Günne J., Levitt M.H. (2001). Double-quantum excitation in the NMR of spinning solids by pulse-assisted rotational resonance. J. Magn. Reson..

[24] Karlsson T., Brinkmann A., Verdegem P.J.E., Lugtenburg J., Levitt M.H. (1999). Multiple-quantum relaxation in the magic-angle-spinning NMR of ^13^C spin pairs. Solid State Nucl. Magn. Reson..

[25] Feng X., Lee Y.K., Sandstrom D., Eden M., Maisel H., Sebald A., Levitt M.H. (1996). Direct determination of a molecular torsional angle by solid-state NMR. Chem. Phys. Lett..

[26] Hong M., Gross J.D., Rienstra C.M., Griffin R.G., Kumashiro K.K., Schmidt-Rohr K. (1997). Coupling amplification in 2D MAS NMR and its application to torsion angle determination in peptides. J. Magn. Reson..

[27] Towse C.L., Rysavy S.J., Vulovic I.M., Daggett V. (2016). New Dynamic Rotamer Libraries: Data-Driven Analysis of Side-Chain Conformational Propensities. Structure.

[28] Gavuzzo E., Mazza F., Pochetti G., Scatturin A. (1989). Crystal-structure, conformation and potential-energy calculations of the chemotactic peptide N-formyl-L-Met-L-Leu-L-Phe-OMe. Int. J. Pept. Protein Res..

[29] Tycko R., Leone S.R., Cremer P.S., Groves J.T., Johnson M.A. (2011). Solid-State NMR Studies of Amyloid Fibril Structure. Annual Review of Physical Chemistry.

[30] Dos A., Schimming V., Huot M.C., Limbach H.H. (2009). Acid-Induced Amino Side-Chain Interactions and Secondary Structure of Solid Poly-L-lysine Probed by ^15^N and ^13^C Solid State NMR and *ab Initio* Model Calculations. J. Am. Chem. Soc..

[31] Middleton D.A. (2024). NMR studies of amyloid interactions. Prog. Nucl. Magn. Reson. Spectrosc..

[32] Petkova A.T., Ishii Y., Balbach J.J., Antzutkin O.N., Leapman R.D., Delaglio F., Tycko R. (2002). A structural model for Alzheimer’s β-amyloid fibrils based on experimental constraints from solid state NMR. Proc. Natl. Acad. Sci. USA.

[33] Madine J., Pandya M.J., Hicks M.R., Rodger A., Yates E.A., Radford S.E., Middleton D.A. (2012). Site-Specific Identification of an Aß Fibril-Heparin Interaction Site by Using Solid-State NMR Spectroscopy. Angew. Chem. Int. Ed..

[34] Scheres S.H.W., Ryskeldi-Falcon B., Goedert M. (2023). Molecular pathology of neurodegenerative diseases by cryo-EM of amyloids. Nature.

[35] Makabe K., Blancalana M., Yan S., Tereshko V., Gawlak G., Miller-Auer H., Meredith S.C., Koide S. (2008). High-resolution structure of a self-assembly-competent form of a hydrophobic peptide captured in a soluble β-sheet scaffold. J. Mol. Biol..

[36] Griffin B.T., O’Driscoll C.M. (2011). Opportunities and challenges for oral delivery of hydrophobic versus hydrophilic peptide and protein-like drugs using lipid-based technologies. Ther. Deliv..

