# Supplementary material for: Conformational Analysis of Uniformly 13C-Labeled Peptides by Rotationally Selected 13Cα-13CH3 Double-Quantum Solid-State NMR
Source: Molecules. 2025 Feb 6;30(3):739. doi: 10.3390/molecules30030739 (PMC11820148; doi:10.3390/molecules30030739)
Supplement: Supplementary file 1 [file molecules-30-00739-s001.zip › molecules-3402829-supplementary.pdf]

Supplementary information for :

Conformational analysis of uniformly-^{13}C-labeled peptides by rotationally-selected $^{13}C\alpha$-$^{13}CH_3$ double quantum solid-state NMR

David Middleton

Department of Chemistry, Lancaster University, Lancaster LA1 4YB, United Kingdom.

Tel: +44 1524 594328

E-mail: d.middleton@lancaster.ac.uk

Contents

Supplementary Tables S1-S3.

Supplementary Figures S1-S9.

Table S1. Summary of ^{13}C chemical shifts and full widths at half height (FWHH) measured from the 1D ^{13}C CP-MAS NMR spectrum of solid [U-^{13}C,^{15}N]fMLF. Chemical shifts were referenced to the ^{13}C signals of adamantane. N.R. = not resolved.

Amino acid	Carbon site	Chemical shift (ppm)	FWHH (Hz)
Met	Cα	54.7	108
	Cβ	43.5	115
	Cγ	31.4	83
	Cε	16.9	47
Leu	Cα	59.8	122
	Cβ	39.6	120
	Cγ	27.9	N.R.
	Cδ	27.7	N.R.
	Cδ′	22.4	67
Phe	Cα	57.1	125
	Cβ	40.6	107

Table S2. Summary of the ^{13}C – ^{13}C DQ coherences selected for solid [U-^{13}C,^{15}N]fMLF, and corresponding MAS frequencies. Numerical values are the MAS frequencies (in Hz) required to meet the *n* = 1 RR condition for each selected spin pair.

	**Mα**	**Mε**	**Lα**	**Lδ′**	**Fα**
**Mα**		6642		5682	
**Mε**	6642		7551		7078
**Lα**		7551		6584	
**Lδ′**	5682		6584		6108
**Fα**		7078		6108	

Table S3. Summary of statistically restricted torsional angles used in the conformational grid search. Allowed values are given as means (standard deviations) of a normal distribution.

Torsional angle number	Atoms involved	Conventional definition	Allowed values (degrees)
1	Cε (Met)–Sδ–Cγ–Cβ	χ_3	311 (3), 60 (5), 169 (3)
2	Sδ–Cγ–Cβ–Cα	χ_2	68 (11), 180 (5), 294 (7)
3	Cγ–Cβ–Cα–C′	undefined[a]	63 (9), 193 (10), 297 (6)
4	Cβ (Met)–Cα–C′–N (Leu)	undefined[b]	55 (25), 250 (50)
5	Cα (Met)–C′–N–Cα (Leu)	ω	180 (10)
6	C′–N–Cα–Cβ	undefined[c]	150 (45)
7	N–Cα–Cβ–Cγ	χ_1	64 (13) ,199 (13) ,291 (12)
8	Cα–Cβ–Cγ–Cδ′	χ_2	59 (5) ,180 (4), 300 (15)
9	N (Leu)–Cα– C′–N (Phe)	ψ	90 (60), 310 (20)
10	Cα (Leu)–C′–N (Phe)–Cγ	ω	180 (10)
11	C′ (Leu)–N–Cγ–C′ (Phe)	ϕ	240 (60)

[a]Corresponds to χ_1 (Cγ–Cβ–Cα–N) plus ~120°.

[b]Corresponds to ψ (N (Met)–Cα–C′–N (Leu)) minus ~120°.

[c]Corresponds to ϕ (C′(Met)–N–Cα–Cβ (Leu)) minus ~120°.

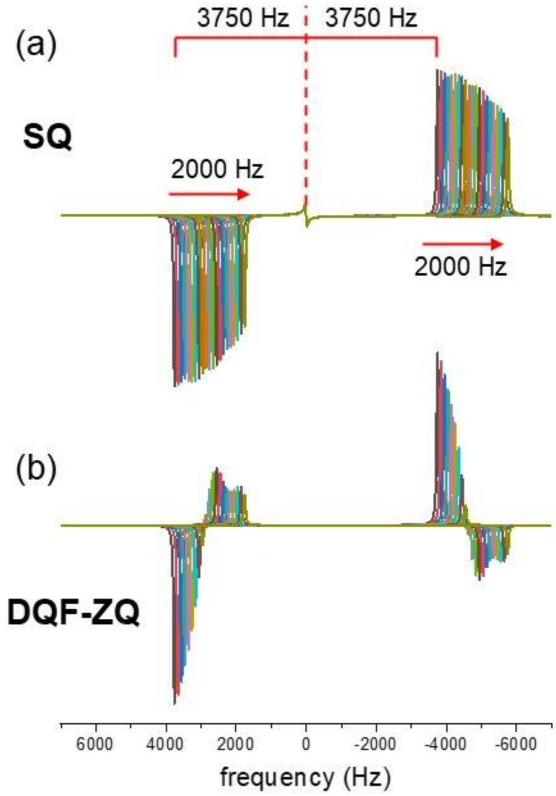

Figure S1. Simulated peak intensity profiles of a dipolar-coupled I–S spin pair (d_{CC} = 180 Hz), showing the effect of varying the resonance frequencies relative to the transmitter/carrier frequency at 0 Hz (dotted line). The pairs of peaks are in each case separated by the MAS frequency of 7500 Hz, i.e., the n = 1 rotational resonance condition. (a) Simulated single quantum (SQ) spectra corresponding to excitation with a $\pi/2_x$–δ–$\pi/2_y$ pulse sequence (where $\delta = 0.5t_R$) to generate I_z–S_z polarization, followed by a $\pi/2_x$ readout pulse. The resonance frequencies of the I–S spin pair increase in 100 Hz increments up to a maximum of 2000 Hz from their original frequencies of ±3750 Hz from the carrier frequency. (b) Simulated spectra at n = 1 rotational resonance, as achieved using the DQRR pulse sequence in Figure 2a of the main text with t_{ev} = 0 and constant t_{ex} = 4 ms. The resonance frequencies vary as described for (a).

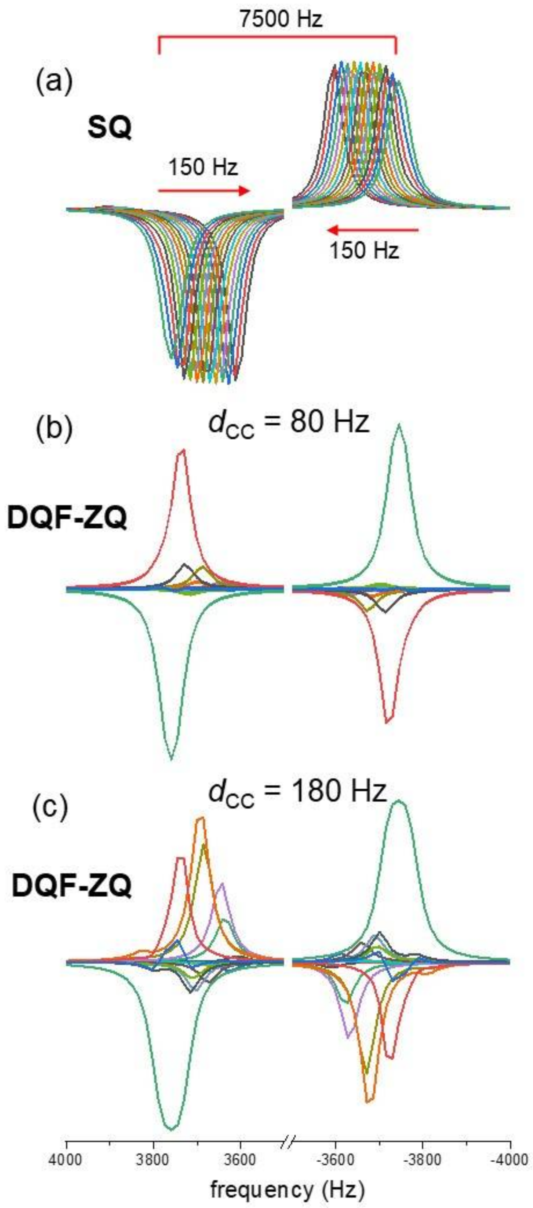

Figure S2. Simulated peak intensity profiles of a dipolar-coupled I–S spin pair, showing the effect of frequency offsets from the n = 1 rotational resonance condition. (a) Simulated single quantum (SQ) spectra. The maximum separation of the peaks is 7500 Hz, which in the simulation corresponds to the n = 1 rotational resonance condition. The separation of the resonance frequencies of the I–S spin pair decreases in 10 Hz steps to a minimum of 150 Hz less than the rotational resonance condition. In each case, both peaks are equally spaced from the carrier frequency at 0 Hz. (b) Simulated spectra at n = 1 rotational resonance, as achieved using the DQRR pulse sequence in Figure 2a of the main text with t_{ev} = 0 and t_{ex} = 4 ms.. The resonance frequencies vary as described for (a). The dipolar coupling constant d_{CC} = 80 Hz. (c) As for (b), but with d_{CC} = 180 Hz.

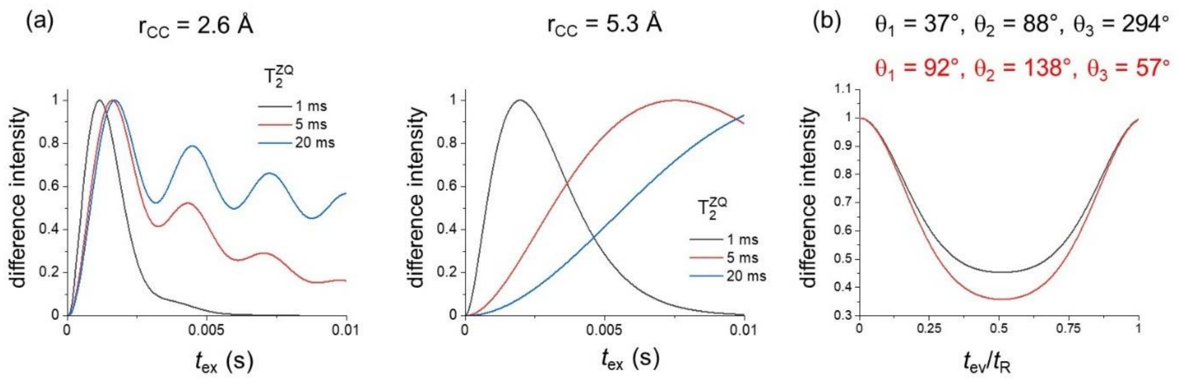

Figure S3. Simulated difference intensity profiles for the DQRR-CC and DQRR-HLF experiments for specific ^{13}C - ^{13}C distances (r_{cc}) and CαH – CH_3 orientations. (a) Difference intensity profiles representing the excitation of ZQ coherence in the VT-DQRR-CC experiment at three different values of the zero quantum relaxation time, T_2^{ZQ}. The left panel corresponds to an internuclear distance, r_{CC}, of 2.6 Å (d_{CC} = 432 Hz) and the right panel corresponds to an internuclear distance of 5.3 Å (51 Hz). (b) Difference intensity profiles representing the evolution of DQ coherence in the unamplified DQRR-HLF experiment.

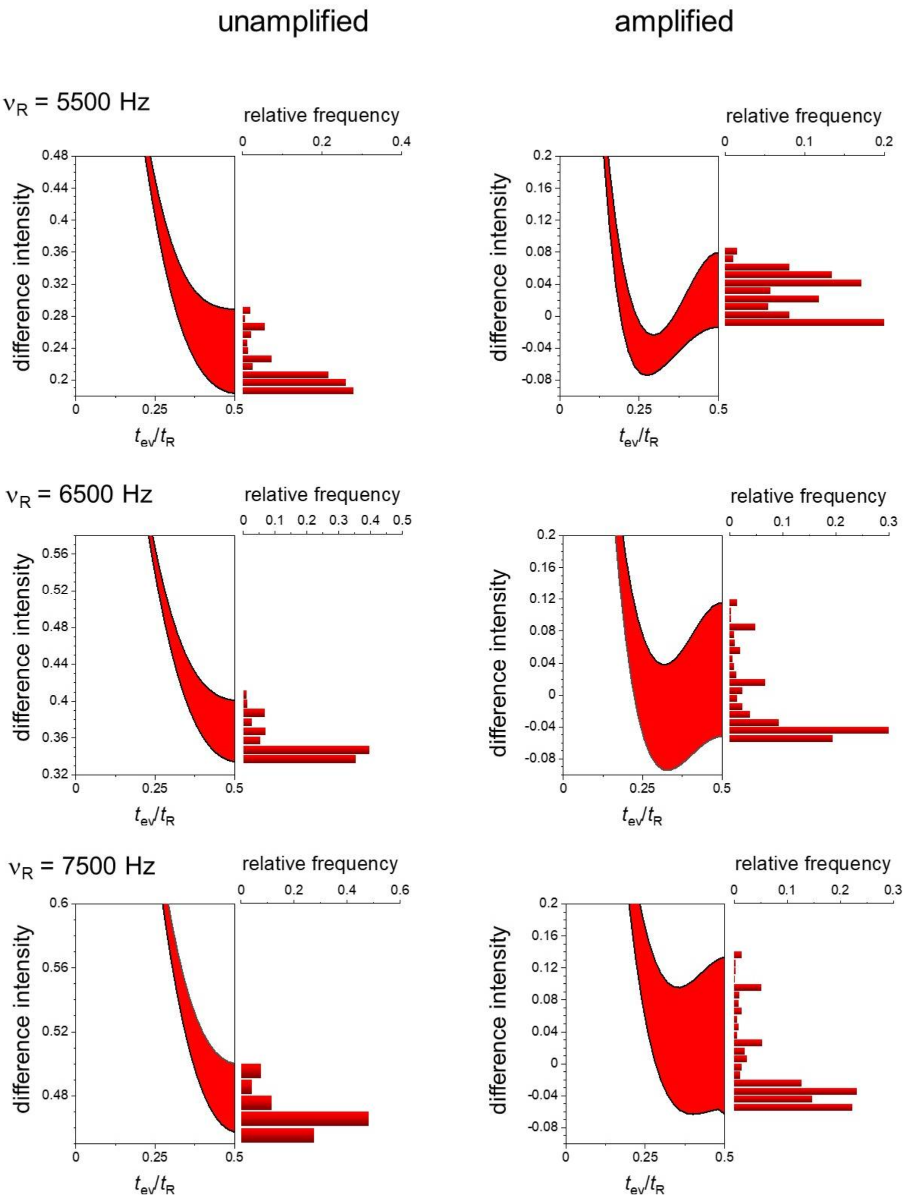

Figure S4.Sensitivity of the DQRR-HLF experiment to the relative orientations of C – H and – CH_3 groups at three difference MAS frequencies. The left panels show calculations for unamplified C – H/CH_3 dipolar couplings (using the pulse sequence in Figure 2a). The right panels show calculations based on the amplified C – H/CH_3 dipolar couplings (using the DQRR-HLF pulse sequence in Figure S4). Within each panel, the left hand section shows the calculated evolution of DQ coherence (measured experimentally as difference magnetization) over the first half of a rotor cycle. DQ evolution over the second half of the rotor cycle is the mirror image of evolution in the first half. The shaded red area indicates the range spanned by the curves for all possible values of effective bond angles θ_1, θ_2 (0 – 180°) and effective torsional angle θ_3 (-180° – 180°). The right hand section of each panel is a histogram indicating the relative frequency of possible [θ_1, θ_2, θ_3] combinations (binned in 0.01 DQ intensity units) that give rise to the DQ intensity at the half rotor cycle point.

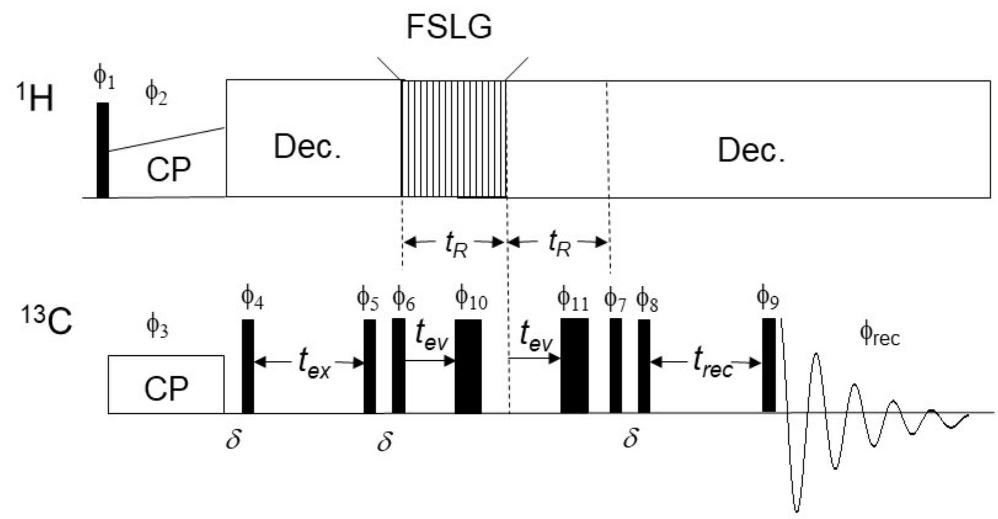

Figure S5. Pulse sequence for the amplified DQRR-HLF experiment. Delays δ, t_{ex}, t_R and t_{rec} are as defined for the pulse sequence given in Figure 2a of the main text. Narrow filled rectangles represent $\pi/2$ pulses and wider filled rectangles represent π pulses. The delay t_{ev} is the only variable in the pulse sequence. A series of spectra is obtained for different values of t_{ev}, each spectrum corresponding to an incrementation of t_{ev} from zero to a maximum of t_R. The π pulses are omitted at both extremes of t_{ev}. Phase cycling: pulse phases $\theta_1 - \theta_9$ and receiver phase ϕ_{rec} are as described for Figure 1b in the main text. Pulse phases ϕ_{10}= +y and ϕ_{11}= +y.

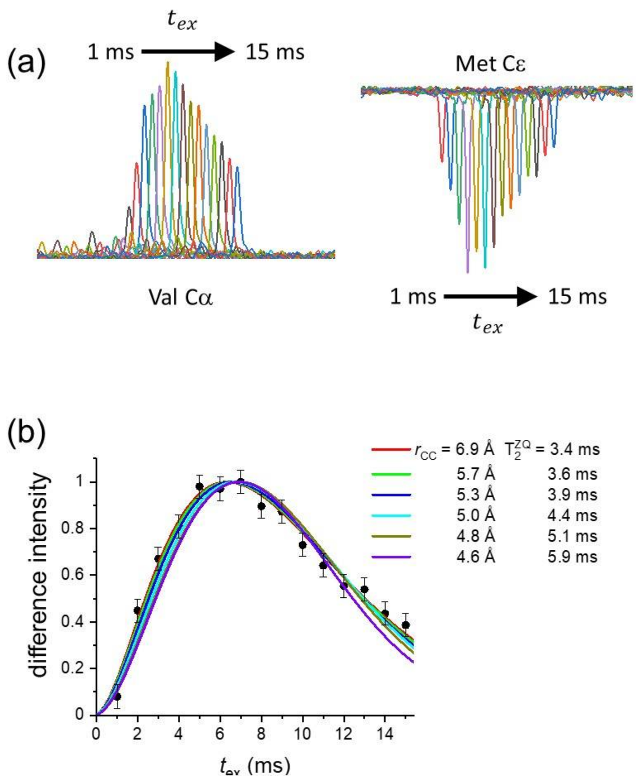

Figure S6. Measurement of the distance-dependent ^{13}Cα – ^{13}Cε dipolar coupling between Leu and Met of solid [U-^{13}C,^{15}N]fMLF, using the VT-RRDQ-CC experiment described in Figure 2a. The MAS frequency was set to the n = 1 rotational resonance with respect to Leu Cα and Met Cε (7551 Hz). In the variable-time experiment, t_{ex} = t_{rec}. (a) Series of spectra obtained by varying t_{ex} and t_{rec}. (b) The difference intensities as a function of t_{ex}, measured from the spectra in (a), and simulated curves for different internuclear distances, r_{CC}, and zero quantum relaxation times, T_2^{ZQ}.

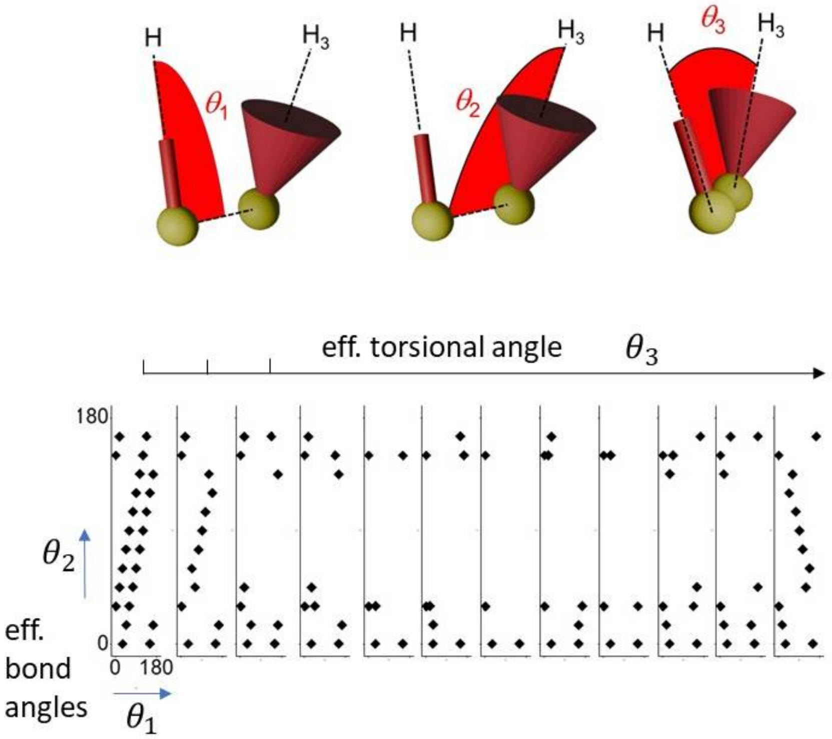

Figure S7. Allowed Cα – H and CεH₃ orientations of Leu and Met in solid [U-^{13}C,^{15}N]fMLF, consistent with the DQRR-HLF measurements shown in Figure 3 (c and d) of the main text.

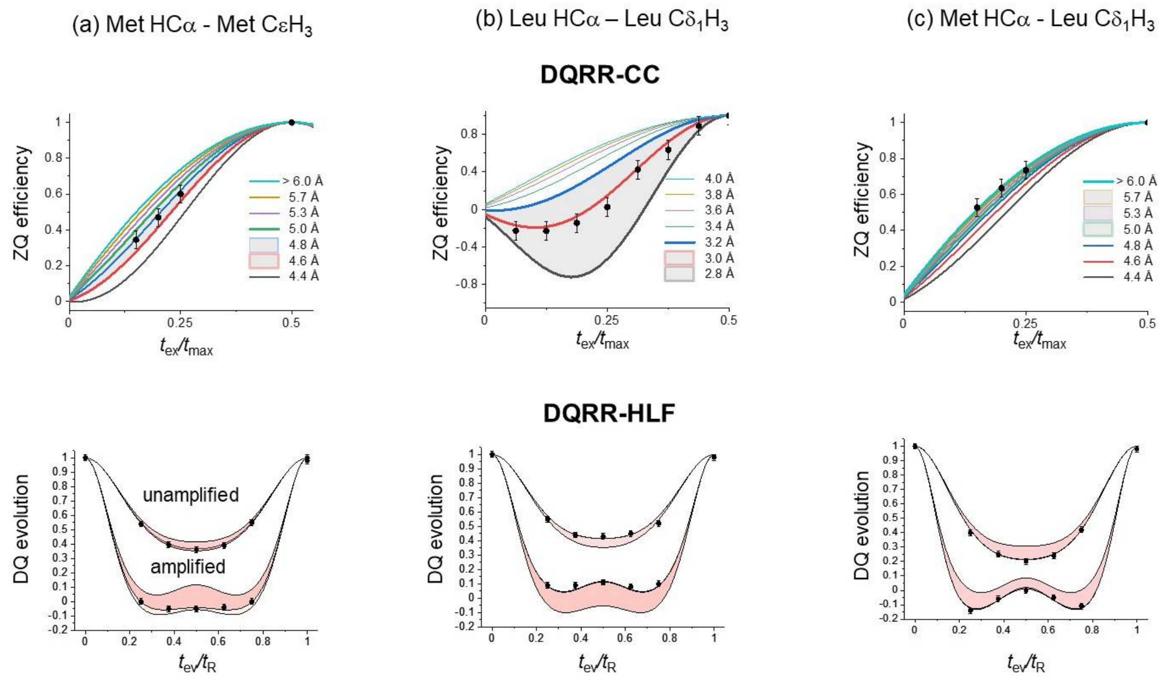

Figure S8. Analysis of the Met side chain conformation in solid [U-^{13}C,^{15}N]fMLF peptide. (a) Cα and aliphatic side chain region of the 1D ^{13}C CP-MAS NMR spectrum, with resonance assignments. (b) 2D RRDQSQ spectra at *n* = 1 RR with respect to the Met Cα and Met Cε resonance frequencies (ν_R = 6642 Hz) and at the excitation times t_{exc} shown. This spectrum and all subsequent spectra were obtained at a magnetic field of 16.4 T. The red arrow denotes the spectrometer transmitter/carrier frequency. (c) ZQ excitation profile and DQ evolution profile measured from the RRDQSQ spectra. Solid lines represent the lines of best fit, obtained as described in the main text. Protons in red are included in the simulations.

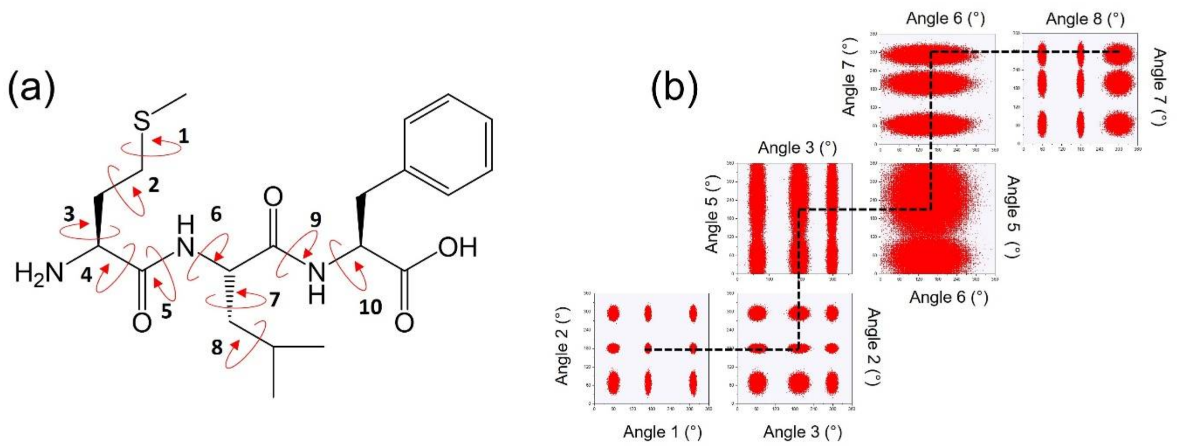

Figure S9. Summary of statistically-weighted probabilities of torsional angles that were varied randomly in the conformational grid search. (a) Definition of torsional angles. (b) The values of the angles generated were weighted according to preferred backbone and side-chain rotamer conformations (see also Table S2). Raw statistical data on conformational preferences of specific amino acids was obtained from https://github.com/jgaines42/MEDFORD-rotamer-library and fitted with multiple Gaussian functions that were used to weight the randomly-generated angles. The dotted line shows one possible peptide conformation that was possible before the SSNMR restraints were applied. Angle 5 (peptide bond angle ω) is omitted, but was assumed to be close to the expect value in peptides and proteins of 180° ± 10°.

```
spinsys{
channels 1H 13C
nuclei 13C 13C 1H 1H
shift 1 0 0 0 0 0 0
shift 2 0 0 0 0 0 0
dipole 1 2 0 0 0 0
dipole 1 3 -11500 0 101 0
dipole 2 4 -3800 0 53 -8
}
par{
method gcompute
spin_rate 7500
gamma_angles 51
sw spin_rate*gamma_angles
np 52
crystal_file rep320
start_operator I1p*I2p+I1m*I2m
detect_operator I1p*I2p+I1m*I2m
}
proc pulseq {} {
maxdt 5.0
delay 1e6
}
proc main {} {
global par
set f [fsimpson]
fsave $f $par(name).fid
}
```

Figure S10. SIMPSON input file for simulations of the unamplified DQRR-HLF curves. The first number highlighted in green corresponds to angle θ_1 in degrees. The numbers highlighted in yellow correspond to 180°- θ_2 and angle θ_3. These angles and the spinning rate were the only variables used in the simulation.

```
spinsys{
 channels 1H 13C
 nuclei 1H 1H 13C 13C


shift 3 0 0 0 0 0 0
shift 4 0 0 0 0 0 0

dipole 1 3 -11500 0 120 0
dipole 2 4 -3800 0 80  180


}

par{
 spin_rate     7500
 gamma_angles   16
 sw          spin_rate*4
 variable tsw   1e6/sw
 variable ni    20
 np          1

 crystal_file   rep320
 start_operator  I3p*I4p+I3m*I4m
 detect_operator 0.125*(I3p*I4p+I3m*I4m)
 proton_frequency 400e6
 verbose      1101
 conjugate_fid  true
 variable rf    250000


}
```

```
proc pulseq {} {
 global par
 maxdt 1.0

 set tr [expr 0.05e6/$par(spin_rate)]
 set t180 [expr 0.5e6/$par(rf)]


 for {set i 1} {$i <= $par(ni)} {incr i 1} {
 reset

 for {set t1 0} {$t1 < $i} {incr t1 1} {
 delay $tr
 }

 pulseid $t180 0 0 $par(rf) x



 for {set t2 $par(ni)} {$t2 > $i} {incr t2 -1} {
 delay $tr
 }
 turnoff dipole_1_3
 turnoff dipole_2_4


 for {set t1 0} {$t1 < $i} {incr t1 1} {
 delay $tr
 }

 pulseid $t180 0 0 $par(rf) x

 for {set t2 $par(ni)} {$t2 > $i} {incr t2 -1} {
 delay $tr
 }


 acq

 }



}

proc main {} {
 global par
  set f [fsimpson]
  fsave $f $par(name).fid

}
```

Figure S11. SIMPSON input file for simulation of the amplified DQRR-HLF curves. This was used as a test file to confirm that the same results were obtained as when doubling the dipolar coupling constants in the file in Figure S10. The file in Figure S10 was then used with doubled dipolar coupling constants for all simulations of the amplified HLD-DQRR curves as it was more computationally efficient.

```
spinsys{
 channels 13C
 nuclei 13C 13C
shift 1 3250 3500 0.69 0 0 0
shift 2 -3250 1750 0.75 0 0 0
dipole 1 2 -200 0 0 0
}

par{
 spin_rate     75000
 gamma_angles   12
 sw           spin_rate*4
 variable tsw   1e6/sw
 variable ni    104
 np          1

 crystal_file   rep320
 start_operator  Inz
 detect_operator I1z-I2z
 verbose       1101
 conjugate_fid  true
 variable rf    250000
}
```

```
proc pulseq{}{
 global par
 maxdt 1.0

 set t90 [expr 0.25e6/$par(rf)]
 set t180 [expr 0.25e6/$par(rf)]
 set tr1 [expr 0.5e6/$par(spin_rate)]


 matrix set 1 notelements{{2 3}{3 2}}
 matrix set 2 notelements{{1 4}{4 1}{1 1}{2 2}{3 3}{4 4}}
 matrix set 3 elements{{1 1}{2 2}{3 3}{4 4}}

 reset
 delay $tr1
 delay $tr1
 store 1

 reset
 delay $tr1
 pulseid $t90 $par(rf) x
 delay $tr1
 pulseid $t90 $par(rf) y

 store 2

 reset
 delay $tr1
 delay $tr1
 pulseid $t90 $par(rf) x
 delay $tr1
 pulseid $t90 $par(rf) y
 delay $tr1

 store 3

 reset
 pulseid $t90 $par(rf) x
 store 4

 for{set i 1}{$i <= $par(ni)}{incr i 1}{
 reset

 prop 2
 filter 3
 for{set t1 0}{$t1 < $i}{incr t1 1}{
 prop 1
 }
 prop 2
 filter 1
 prop 3
 filter 2
 for{set t2 $par(ni)}{$t2 >= $i}{incr t2 -1}{}
 prop 1
 }
 prop 4

 acq

 }

}

proc main{}{
 global par
  set f [fsimpson]
  fsave $f $par(name).fid

}
```

Figure S12. SIMPSON input file for simulations of the CT-DQRR-CC curves. Chemical shift tensor values (green) were averaged from various literature sources. Euler angles defining the tensor orientations (yellow) were kept at zero. In practice, nonzero values of these angles had little effect on the simulated curves.
